# Dissolution of Portlandite in Pure Water: Part 1 Molecular Dynamics (MD) Approach

**DOI:** 10.3390/ma15041404

**Published:** 2022-02-14

**Authors:** Khondakar Mohammad Salah Uddin, Mohammadreza Izadifar, Neven Ukrainczyk, Eduardus Koenders, Bernhard Middendorf

**Affiliations:** 1 Department of Structural Materials and Construction Chemistry, University of Kassel, Mönchebergstraße 7, 34125 Kassel, Germany; middendorf@uni-kassel.de; 2 Institute of Construction and Building Materials, Technical University of Darmstadt, Franziska-Braun-Str. 3, 64287 Darmstadt, Germany; izadifar@wib.tu-darmstadt.de (M.I.); koenders@wib.tu-darmstadt.de (E.K.)

**Keywords:** cement hydration, dissolution of portlandite, free energy surfaces, surface properties, molecular dynamics simulation, reactive force field, metadynamics

## Abstract

The current contribution proposes a multi-scale bridging modeling approach for the dissolution of crystals to connect the atomistic scale to the (sub-) micro-scale. This is demonstrated in the example of dissolution of portlandite, as a relatively simple benchmarking example for cementitious materials. Moreover, dissolution kinetics is also important for other industrial processes, e.g., acid gas absorption and pH control. In this work, the biased molecular dynamics (metadynamics) coupled with reactive force field is employed to calculate the reaction path as a free energy surface of calcium dissolution at 298 K in water from the different crystal facets of portlandite. It is also explained why the reactivity of the (010), (100), and (1$\bar{1}$0) crystal facet is higher compared to the (001) facet. In addition, the influence of neighboring Ca crystal sites arrangements on the atomistic dissolution rates is explained as necessary scenarios for the upscaling. The calculated rate constants of all atomistic reaction scenarios provided an input catalog ready to be used in an upscaling kinetic Monte Carlo (KMC) approach.

## 1. Introduction

Although the crystal dissolution (and precipitation) kinetics are of wide industrial importance, upscaling of fundamental atomistic modeling approaches still presents a major scientific challenge. The dissolution (and precipitation) of minerals phases is governed by complex physico-chemical processes starting at the atomistic scale, where the individual crystal building units (atoms or molecules) at the solid–liquid interface are dissolved and transported into the bulk solution. At mesoscale, e.g., 0.1–10 µm length scale and microsecond time scales, of interest is to predict the evolution of the crystal morphology, as a function of the transport processes and concentration effects. At the atomistic scale, molecular dynamics (MD) computations provide a powerful way for revealing how the atomistic processes of basic building units of the crystals affect the dissolution kinetics. This paper focuses on the atomistic models for the dissolution of crystals. To close the long south bridging gap between the atomistic and the meso-scale modeling approaches for cementitious materials, far-from-equilibrium conditions were considered. Therefore, the dissolution of portlandite (CH, Ca(OH)_2_), was selected as a relatively simple crystal representative for the dissolution/precipitation process of other more complex cementitious phases (e.g., cement clinker phases) in general. Portlandite is a key by-product of cement hydration in the amount of approximately 27% in volume, which protects the steel reinforcement from corrosion by maintaining a higher pH value [1].

Portlandite dissolution leads to carbonation that accelerates the corrosion of the reinforcement by dropping the pH value of the pore solution by approximately three units [2,3,4]. During the hydration of cement, portlandite does not only precipitated in the bulk of the hardened cement paste matrix but also fills in a more porous interfacial layer between the steel reinforcement and aggregate [5]. Besides its significance in building materials, portlandite is also used in environmental applications such as (flue) acid gas absorption in wet scrubbers, spray dryer reactors, and for pH control of wastewaters. Portlandite can also be used to reduce the carbon footprint as well as thermochemical energy storage [6,7].

Due to portlandite’s relatively simple crystal structure, we propose it as a benchmarking mineral phase in developing atomistic modeling approaches for the dissolution/precipitation process of other (more complex) cementitious minerals in general. The dissolution of cementitious minerals, especially at an atomistic level, is not fully understood yet due to the lack of experimental and modeling techniques available to reach this resolution and bridge the time and scale gaps. Within the past decade, many atomistic-level computational methods have been developed to describe cementitious materials. Density functional theory (DFT) using quantum mechanics is known to be comparatively accurate for geometry optimization [8], vibrational energy [9], band structure calculation [10], mechanical, electronic, and optical properties of the small molecule [11,12]. However, this method is highly computationally expensive and practically not applicable to a large system. On the other hand, classical force field (FF) theory is unable to explain the bond formation and bond breaking. Though Brenner FF theory is able to describe bond-breaking, the Coulomb interactions are not considered [13]. Besides, the bond-energy bond-order (BEBO) method could not explain more complex reactions. Therefore, atomistic simulation approaches using reactive force fields (ReaxFF) parameterized by quantum mechanical calculation, in combination with metadynamics (metaD), have been proposed as a potential solution to study the chemical reactions pathways with satisfactory accuracy and reasonable computing times.

ReaxFF has been developed to elucidate the interfacial reaction mechanism. It has been implemented successfully in hydrocarbons [14], polymer chemistry, metal oxides (Si/SiO_2_) [15,16], metal hydrides [17], and many other systems. ReaxFF calculates the molecular dynamics in femtosecond (i.e., 10^−15^ s) time steps for the correct integration of equations of motion. Although the ReaxFF is much more efficient compared to traditional classical force field approaches [18], sometimes it becomes computationally expensive during simulations of interesting events (e.g., atomistic transition states) that typically occurred in a longer time scale. To solve this timescale issue, metadynamics (metaD) is combined with ReaxFF, enabling a powerful coupling that can accelerate the reaction by adding a biased potential that acts on an appropriate number of degrees of freedom usually known as collective variables (CVs) (i.e., inter-atomic/molecular distance, co-ordination number, angles, etc.). During the MD simulation, the bias potential is applied as a sum of Gaussian, continuously growing and acting directly on the localized coordinates of the system [19].

The combined approach has already shown a great potential to calculate the dissolution mechanism and the reactivity of different facets of Portland cement clinkers at the atomistic scale [20]. The current contribution aims at the development of a multi-scale modeling approach that links the atomistic scale to the (sub-)micro-scale. It will open up new insights into the reaction kinetics of portlandite dissolution, and establish a benchmark approach for cementitious materials in general.

In this research (Part 1), a multistep modeling approach has been taken to achieve a clear overview of the dissolution and reactivity of different facets of Portlandite at room temperature (298 K). First, pre-simulations were run to allow for pre-hydration of (001), (100), (010) facets of portlandite, for 600 picoseconds. Then, all pre-hydrated facets were used as an initial geometry to study one-by-one dissolution mechanisms of calcium atoms by using a combination of ReaxFF and metaD.

The reactivity of different crystal facets was compared with the dissolution profile (free energy surface) of calcium. The orientation of calcium and the number of (crystal site) neighbors are different on each crystal facet and change with dissolution progress due to removal of the neighbors. This creates many possibilities which can take place and are called here dissolution scenarios. Therefore, the influence of crystal site neighbors is considered here as a critical step in upscaling the individual atomistic simulations into a meso-scale simulation. Finally, the results are used to calculate the atomistic rate constants using a transition state theory (TST). For this, the most critical possible events have to be identified and quantified to be provided as an input for upscaling using a kinetic Monte Carlo (KMC) approach (part 2 paper) and calculating the overall rate of the dissolution and morphological changes [21].

## 2. Methods and Modeling Approach

The ReaxFF has been employed successfully in the cementitious system by merging two parameter sets (Si-O-H and Ca-O-H) developed by Fogarty et al. [22] and Manzano et al. [16], individually. This parameter set has already explained the absorption of water to C_3_S clinker phases [20]. The computer simulations were carried out by using reactive force field theory (ReaxFF) in LAAMPS (Large-scale Atomic/Molecular Massively Parallel Simulator) platform [23].

Usually, in a force field MD simulation, the femtoseconds (10^−15^ s) time step is considered. However, the majority of the interesting phenomena (i.e., transition state events (TS)) occur at a much larger time scale. Therefore, the rare event calculation requires millions of time steps, which increases the computational cost. Therefore, many sampling methods have been developed to reduce the computation time.

Metadynamics is a powerful algorithm applied for the free energy calculation by accelerating the rare event sampling. In metaD, a history dependent external biased potential as a function of CVs is imposed in a controlled manner to enhance the exploration of free energy surface (FES). The potential is added along with the CVs space as a sum of Gaussians, which encourages the system to visit the unexplored configurations. After prolonging the simulation for a sufficiently long time, the biased potential is converged to reconstruct the FES as a function of that selected CVs [24].

### Model Construction

The Portlandite is a trigonal layered crystal structure with space group $P\bar{3}m$. Each hydroxyl group connects three Ca atoms in its (001) layer and is also surrounded by three other hydroxyl groups from the next (001) layer. In this work, a hexagonal Portlandite crystal model was used with the lattice parameter of a = b = 3.586 Å, c = 4.911 Å, α = β = 90°, and γ = 120° [25]. The fresh cleaved (001) Portlandite orthogonal periodic simulation cell (17.80 × 19.64 × 38.65) × 10^−30^ m^3^ contained 1302 atoms was built using virtual nano lab (VNL) [26] and Avogadro [26,27]. The geometry of the simulation cell was optimized using Hessian-free truncated Newton algorithm (HFTN) where the stopping tolerances for energy were 1.0 × 10^−4^ and force was 1.0 × 10^−6^ kcal mol^−1^ Å^−1^ [28]. Afterward, 6.99 × 10^−27^ m^3^ periodic cell filled with randomly distributed water molecules (density 1000 kg m^−3^) was added up to the optimized (001) Portlandite facet using packmole [29]. First, the simulation cell was equilibrated for 150 picoseconds (0.5 femtoseconds time steps) at 298 K and 1 atm using Nose−Hoover thermostat (NVT, canonical ensemble. Later, the equilibrated geometry was hydrated for 600 picoseconds using Nose−Hoover barostat (in NPT canonical ensemble). A periodic boundary condition was applied during the entire simulation.

The hydrated (001) facet of Portlandite (after 600 picoseconds) was taken as an input geometry to compute the dissolution mechanism of calcium using the combined metaD and ReaxFF.

**Figure 1:**
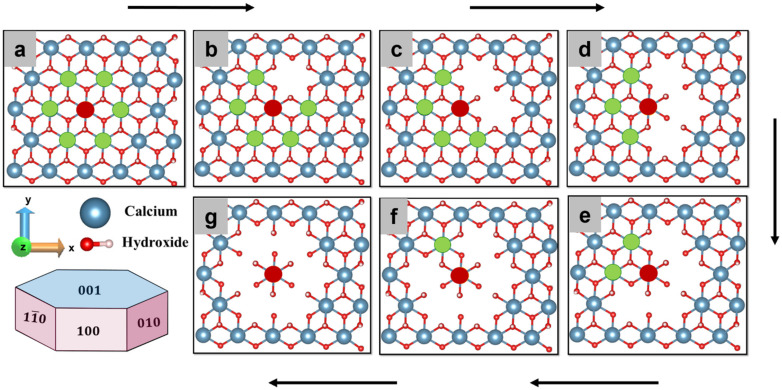
Dissolution scenarios of hexagonally oriented central Ca (red) from (001) facet of Portlandite (**a**) in the presence and (**b**–**g**) absence of 1,2,3,4,5,6 neighbors (green) removed one by one (depicted in a clockwise manner).

The PLUMED [30,31] package as an extension of LAMMPS [23] was employed for metaD simulation. It speeds up the simulation using history-dependent biased potential. A calcium Ca-588 from (001) facet located in the center of hexagonally oriented neighboring Ca (Figure 1a) of the hydrated Portlandite facet is selected. A well-tempered metaD scheme was applied to remove calcium from the crystal facet into the pore solution. The distance between the center of mass (COM) of the crystal and Ca-588 is selected as a CV. It is worth noting that the choice of the correct CVs is crucial for getting a good physical description. The bias potential was added as a Gaussian with a height of 6.28 kJ/mol and full width at half-maximum of 0.2 × 10^−10^ m in every 0.02 picoseconds.

The simulation was performed for 300 picoseconds (until convergence) at 298 K with NPT ensemble and the free energy of dissolution was computed over the entire. In order to understand the influence of hexagonally oriented Ca neighbors, the identical approach was applied to compute the dissolution profile of central Ca (red) in absence of 1,2,3,4,5,6 neighbor individually being pre-deleted in a clockwise manner (Figure 1b–g). In this manner, the activation barrier for a total of seven scenarios was calculated to investigate the influence of neighbors (scenario) configurations that are expected to occur during the (longer-term) dissolution. Those scenarios are of key relevance for upscaling, e.g., to provide required inputs for kinetic Monte Carlo simulations.

**Figure 2:**
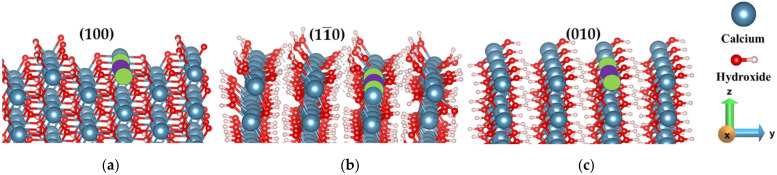
Dissolution scenarios of central Ca (violet) in absence of first and second nearest neighbors (green) removed one by one from: (**a**) (100), (**b**) (1$\bar{1}$0) and (**c**) (010) facet of Portlandite.

A similar approach was used for other crystal facets: (100), (010), and (1$\bar{1}$0) cleavage facets of portlandite (Table A1), where after the pre-hydration simulations, the possible (most relevant) scenarios for the dissolution mechanism are simulated. The reactivities were calculated from the dissolution profile (FES) of the calcium of the individual facet. Differently to the (001) facet, where the neighbor Ca is arranged in a hexagonal pattern, the Ca in on (100), (1$\bar{1}$0), and (010) facets are arranged in a row (linear, i.e., left and right) manner). Therefore, a total of three scenarios were considered to calculate the dissolution behavior of the central Ca before and after removing 1 and 2 neighboring Ca located on both sides (marked green) in the same row (Figure 2). In addition, there is one neighbor below (direction-z) which cannot be dissolved before the central atom.

Finally, this study delivers a complete set of all (most important) events for the four different facets of portlandite, relevant for a hexagonal crystal morphology (Figure 1). Results of these simulations provide energy barriers, which are exponentially proportional to the individual rate constant of all individual events, e.g., according to transition state theory (TST). A full catalog of individual (atomistic) reaction rates provided basic input of upscaling KMC simulation for calculating the overall rate of the dissolution and morphological changes (published separately as part 2 paper to this Issue).

## 3. Results and Discussion

### 3.1. Pre-Hydration of Portlandite

In order to understand the interaction between bulk water and Portlandite facet, the dynamics of the system have been followed for 600 picoseconds at 298 K and standard ambient pressure (101.325 kPa). The periodic boundary condition was applied to all three directions to improve the representativeness of the elementary volume cube size during simulation. Hence, when an atom passes one side of the cell, it reappears on the opposite side with the same velocity. A periodic dimension can change in size due to constant pressure boundary conditions. In addition, 0.5 fs (femtosecond) time steps were used for the entire simulation to accurately capture the fastest vibrations of hydrogen, as the smallest and lightest element in the system. Moreover, ReaxFF can be considered the smallest atoms as bonded only for sufficiently small time steps.

According to our observation, at the very initial stage, the water molecules interact with the facets of Portlandite and the charge transfer between the O atom (part of the hydroxyl group) in the first layer of (010) facet and the H ion of the water molecule is comparatively more obvious than the charge transfer between Ca and the O (water) atoms. Therefore, the electrophilic attack is much stronger than the nucleophilic attack during water adsorption on the (010) facet. Afterward, the proton transfer occurs from the hydroxyl of the surface to the inner oxygen by leaving the first oxygen-free for further reaction (hopping process) as described by Manzano et al. [32]. Pre-hydration is carried out only for 600 picoseconds.

**Figure 3:**
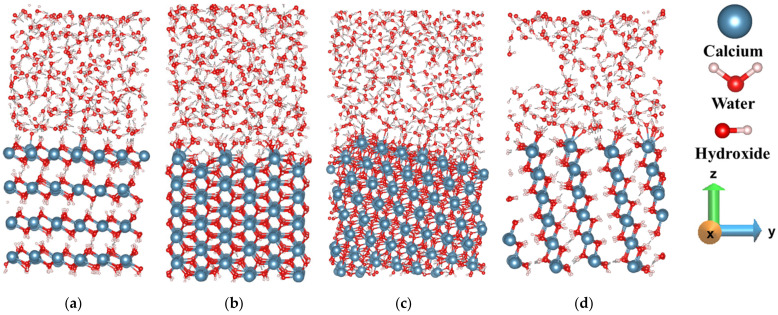
Comparison of the reactivity between the different facets of Portlandite: (**a**) (001), (**b**) (100), (**c**) ($1\bar{1}0$), and (**d**) (010). Snapshots after pre-hydration simulations for 600 picoseconds at 298 K.

The (010) facet (prismatic facet) of Portlandite (Figure 3d) shows higher reactivity during hydration compared to the basal facets (001). The main reason is that the atoms on the (010) facet can be the electrophilic and nucleophilic center, therefore, the charges were balanced more significantly, resulting in lower surface energy. This indicates the reason for the higher interaction between bulk water and the facet, resulting in the formation of voids (Figure 3d). Similarly, (100) and ($1\bar{1}0$) facets also exhibit such behavior at an intermediate level, indicating intermediate reactivity (Figure 3b,c).

In contrast, the (001) crystal facet indicates the lowest reactivity due to their highest surface energy and water tessellation during pre-hydration. The term ‘water tessellation’ describes the arrangement of the water molecule as a quasi-static 2D sheet barrier [33,34]. The water tessellation at the (001) facet prevents water from penetration into the crystal and the dissolution of calcium ions from the facet (Figure 3a).

Traditionally, the reactivity of surfaces was attempted to correlate with their static properties, i.e., surface energies. However, the results of dynamic pre-hydration by MD simulations at the interface between the Portlandite and water, demonstrate the lack of such a correlation. Generally, the facet properties are modified during the interaction of water. Therefore, only a correlation of the facet properties is insufficient. Hence, the reactivity of portlandite facets depends on the interfacial reaction. The free energy calculation during the dissolution of Ca from the different facets of portlandite to the solution would provide an appropriate explanation about the reactivity.

### 3.2. Dissolution of Calcium from (001) Facet of Portlandite

Free energy surface (FES) calculation has received great interest in MD simulations for obtaining a clear impression of reaction paths, including transition states. Well-tempered metaD is an efficient method that accelerates the chemical reaction to overcome the activation barrier by. Selecting suitable CVs is the main challenge in metaD-based computation. Calculating the FES of a targeted region is the main advantage of metaD. In the current study, the ReaxFF coupled with metaD enabled the calculation of the dissolution pathways of calcium from the facets of portlandite.

**Figure 4:**
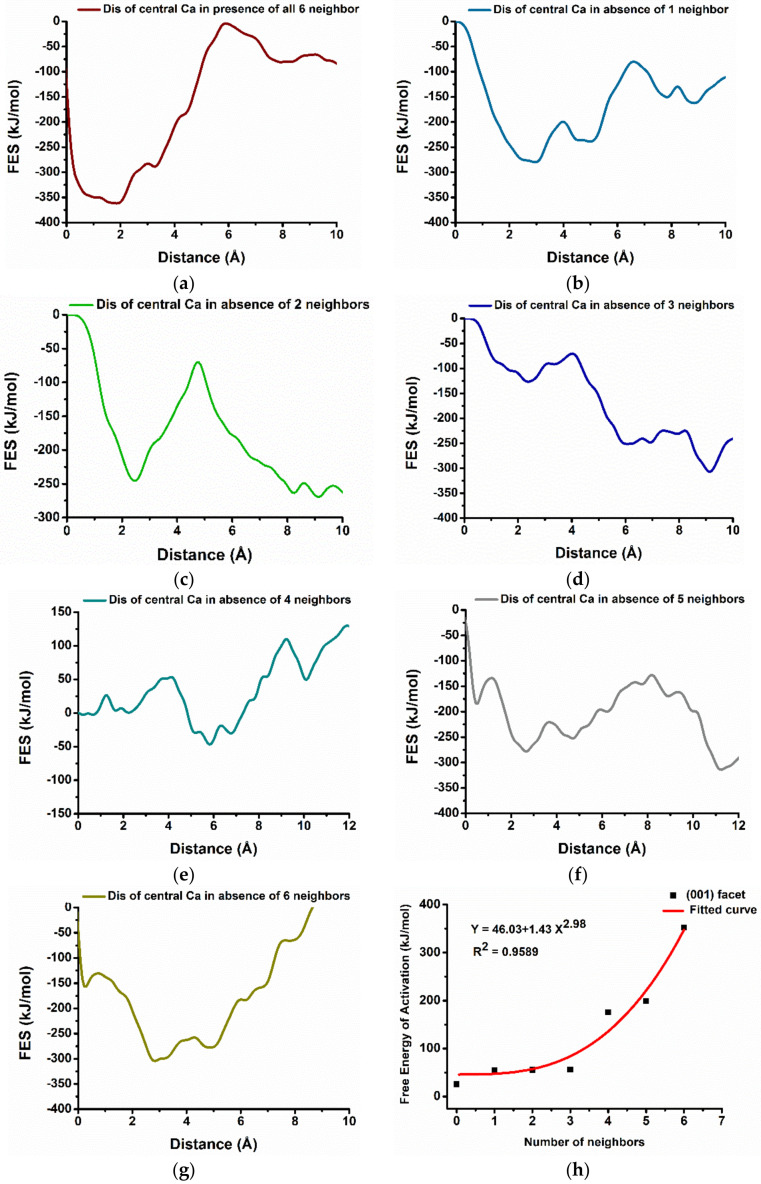
Representative dissolution profile (free energy surface) of the hexagonally oriented central Ca (red Ca in Figure 1) from (001) crystalline facet of Portlandite in different scenarios: the presence of all six Ca neighbors (**a**) and absence of the different number of 1,2,3,4,5,6 (**b**–**g**) hexagonally oriented neighbors. (**h**) The correlation diagram of free energy of activation of central Ca of (001) facet and hexagonally oriented number of Ca neighbors at 298 K.

Figure 4a represents the dissolution profile of calcium from (001) crystalline facet of portlandite at 298 K selecting a single CVs distance between the central Ca-588 surrounded by six Ca neighbors (hexagonally oriented) and the center of mass of the crystal. The x-axis represents the distance (reaction coordinate) in Å (10^−10^ m) starting from the initial state on the facet (0 Å), to the solution (final state) at the next lowest minima.

The free energy surface, from the metaD calculation, represents the movement of Ca-588 from the center of the hexagon to the pore solution required to overcome the huge activation barrier of 352.0 kJ/mol at 5.88 × 10^−10^ m. (Figure 4a). The total free energy change (ΔG) of +280.8 kJ/mol and high activation barrier are directed to the endergonic, i.e., thermodynamically not favorable process and less reactive facets.

Nevertheless, after removing the first and second neighbor clockwise (Figure 1b,c) the activation barrier decreases to 175.4 kJ/mol (still high); however, the dissolution process becomes thermodynamically favorable (ΔG = −23.6 kJ/mol) (Figure 4b,c). Further removal of neighboring Ca one by one reduces the activation barrier significantly for the dissolution of the same central Ca and reaches its minimum value of 25.9 kJ/mol (Table 1) when all six neighbors are missing (Figure 4d–g). The results complement the previous observation, where after 600 ps of pre-hydration, the (001) facet of Portlandite was found less reactive due to stabilizing the facet by its compact geometry and water tessellation [32]. Therefore, it is very difficult to dissolute Ca from the perfect (001) crystal facet; however, the defects, grain boundaries, and missing Ca neighbors (as demonstrated in Figure 4) could enhance its reactivity.

Furthermore, a correlation is done between the seven scenarios of Figure 1 and the resulting Free energy of activation (Table 1). The values of the activation barrier can be fitted as a function of the number of neighbors (*x_i_*) with an allometric (basic Origin) function *y_i_* = a + b*x_i_*^c^, where *a* = 46.03, *b* = −1.43, and *c* = 2.98 as calibrated parameters. The R-square coefficient of 0.958 was indicated that the model follows the trend of the data satisfactorily (Figure 4h).

### 3.3. Dissolution of Calcium from Prismatic (100), (110), and (010) Facets of Portlandite

**Figure 5:**
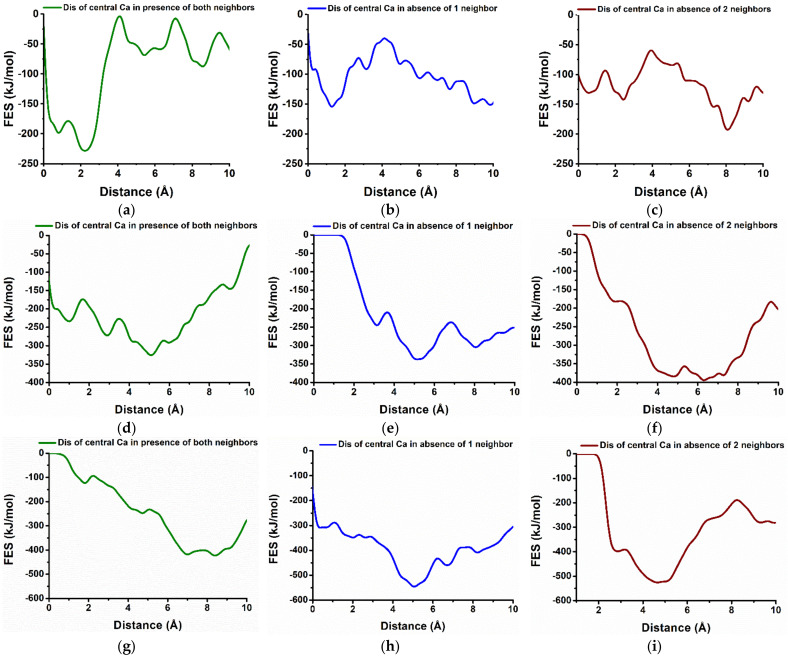
Characteristic dissolution profile (free energy surface) of the central Ca (violet Ca in the Figure 2) (**a**–**c**), from (100); (**d**–**f**); from ($1\bar{1}0$); and (**g**–**i**); from (010); crystalline facets of Portlandite in the different scenarios: before and after removal of one and two neighbors.

The free energy profile of calcium dissolution from the (010) facet shows higher reactivity than (001) in agreement with the results after 600 picoseconds of pre-hydration. The dissolution of Ca-552 from (010) facet was required to overcome the barrier of 29.9 kJ/mol at 1.71 × 10^−10^ m (Table 1). The total free energy change (ΔG) of −299.7 kJ/mol at 298 K indicates an exergonic reaction and thermodynamically favorable [35,36]. This explains the high reactivity and higher interaction of water, so rapidly attracted to the facet that it results in the creation of voids (Figure 3d). Moreover, the activation barrier for the dissolution of the same central calcium (Ca-552) decreased to 20.5 kJ/mol and 7.1 kJ/mol in absence of the first and second neighbor respectively which was removed one by one manner (Figure 5h,i).

Likewise, Ca dissolution (central Ca) from ($1\bar{1}0$) facet of portlandite followed the same (thermodynamically favorable) behavior as (010) facet—however, in a comparatively less reactive fashion, due to the slightly higher activation barrier (56.0 kJ/mol) in the presence of both neighbors, while lower for one neighbor missing (34.9 kJ/mol) (Figure 5d,e). Nevertheless, in absence of both neighbors, the activation barrier was found by far the lowest (0.7 kJ/mol) among all the studied scenarios during the dissolution of central Ca (Table 1).

Among the three prismatic facets of portlandite, the dissolution behavior of the (100) facet resembled the basal facet (001) in terms of facet reactivity and influence of Ca neighbors. The complete dissolution of central calcium (Ca-542) in presence of all neighbors requires 195.3 kJ/mol at 2.22 × 10^−10^ m which is lower than the (001) facet in a similar scenario. The total free energy change (ΔG) of +111.1 kJ/mol at 298 K represents an endergonic and thermodynamically unfavorable process (Figure 5a). The activation energy reduces to 114.6 kJ/mol when one neighbor is missing, still following a similar trend. Furthermore, in absence of both neighbors, the dissolution process become favorable (−62.3 kJ/mol) and the free energy of activation was further reduced to 70.0 kJ/mol which was higher compared to the (010) and ($1\bar{1}0$) facets even in presence of both neighbors (Figure 5b,c).

**Figure 6:**
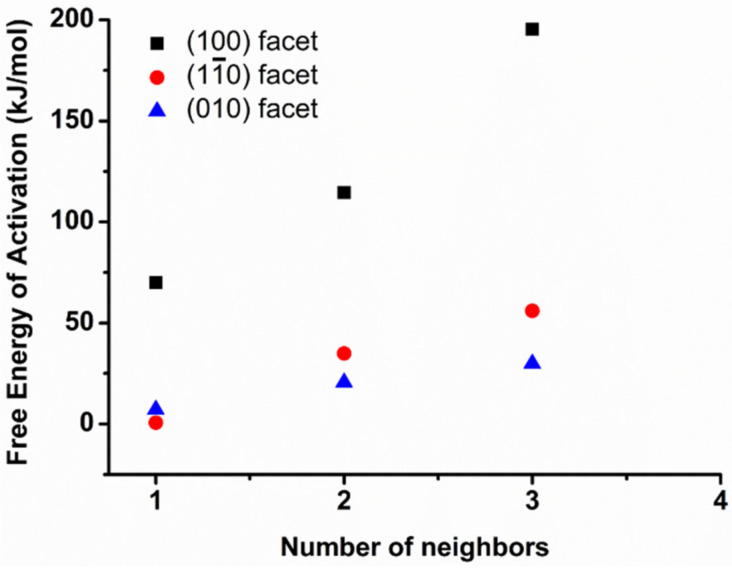
Correlation diagram of free energy of activation of central Ca (violet Ca in Figure 2) of (100), ($1\bar{1}0$), and (010) crystalline facets of Portlandite as a function of number of Ca neighbors at 298 K.

The correlation diagram represents the increase of the activation barrier with increasing the number of neighbors for all three prismatic facets (Figure 6).

### 3.4. Reactivity of Different Facets of Portlandite

Considering the free energy calculation for the different facets of Portlandite in different scenarios, both (001) and (100) facets were not reactive especially in the perfect condition (no missing neighbors); however, they become reactive in absence of neighbors. In contrast, (010) and ($1\bar{1}0$) were found reactive in all cases where the reactivity increases with the number of missing neighbors. Therefore, neighbors played a significant role in the reactivity of the dissolution mechanism.

**Figure 7:**
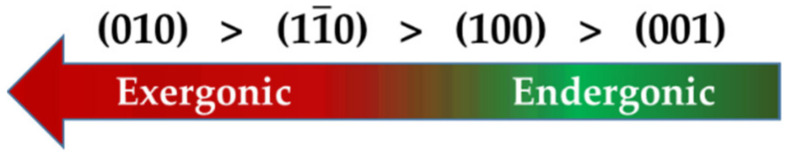
Overall reactivity order of different facets of Portlandite comparing dissolution profile of central calcium in presence of all neighbors (at 298 K).

Finally, the thermodynamic properties for the dissolution of calcium at the perfect crystalline facets, the reactivity order for the different facets of portlandite are shown in Figure 7.

### 3.5. Upscaling the Dissolution Rate for Different Facets of Portlandite

To upscale the atomistic simulations towards much larger timescales and meso-scopic—i.e., sub-micrometer system-sizes, kinetic Monte Carlo (KMC) simulations are applied. The principal idea of a KMC simulation is in bridging the timescale gap by coarse-graining the time evolution and focusing on discrete rare events using Markovian state-to-state dynamics [37]. The calculated activation barrier obtained here by MD simulations (using ReaxFF coupled with MetaD) is tabulated for all scenarios for the dissolution of calcium from the different crystalline facets (Table 1). This table is considered to be the most critical and tedious step for the KMC upscaling model. Results of this paper are provided as input data for KMC simulations in Part 2 publication [38], where the rate of calcium dissolution for the individual scenarios was calculated using transition state theory [39]. Finally, KMC can predict the overall mesoscopic (far-from equilibrium) rate of dissolution and morphological change for any user-defined initial crystal size and morphology.

## 4. Conclusions

The primary objective of this research was to achieve a deeper understanding of the dissolution mechanism of portlandite; therefore, different crystal faces were considered. The upscaling approach was established for the atomistic scale simulation using ReaxFF coupled with the metaD to feed the sub-micro KMC modeling approach. Simulations identified the key dissolution reaction events from possible scenarios depending on nearest neighbors Ca configurations, and the different crystal facets of Portlandite.

The results show that the (010) and ($1\bar{1}0$) facets (prismatic facet) were found most reactive and the dissolution of calcium was thermodynamically favorable. Besides, the lower the number of (dissolved) Ca-neighbors, the lower the activation barrier—i.e., higher atomistic event dissolution rate. In contrast, (001) and (100) were shown the lowest reactivity, and the dissolution was unfavorable at the perfect facet. However, the dissolution rate increased by decreasing the number of (dissolved) Ca-neighbors, which explains that the crystal defects, grain boundaries, and absent neighbors increase the dissolution rate.

The (001) facet was found to be the least reactive facet, where the water tessellation maintained the atomic arrangement of the crystal facets, preventing Ca dissolution. Whereas high interaction of water and lower activation barrier on the dissolution profile of Ca indicated the (010) facet to be the most reactive one.

Finally, the calculated dissolution rate catalog of the most important scenarios is obtained to feed KMC simulations for upscaling computations to predict the mesoscopic dissolution rates and evolution of the crystal morphology.

## Figures and Tables

**Table 1:** Free energy change of different facets of Portlandite during the dissolution of calcium at standard ambient temperature (298 K) and pressure (101.325 kPa).

Portlandite Crystal Plane	Possible Scenarios of Dissolution of Central Ca	Free Energy of Activation ($\Delta G^{*}$) kJ/mol	Free Energy Change (ΔG) kJ/mol	Thermodynamic Properties
(001)	In presence of 6 neighborsΔ	352.0	+280.8	endergonic
	In absence of 1 neighbor	199.1	+117.7	endergonic
	In absence of 2 neighbors	175.4	−23.6	exergonic
	In absence of 3 neighbors	56.1	−180.5	exergonic
	In absence of 4 neighbors	55.8	−44.3	Exergonic
	In absence of 5 neighbors	54.9	−130.0	Exergonic
	In absence of 6 neighbors	25.9	−147.6	Exergonic
(100)	In presence of 2 neighbors	195.3	+111.1	Endergonic
	In absence of 1 neighbor	114.6	+5.6	Endergonic
	In absence of 2 neighbors	70.0	−62.3	Exergonic
($1\bar{1}0$)	In presence of 2 neighbors	56.0	−92.2	Exergonic
	In absence of 1 neighbor	34.9	−59.4	Exergonic
	In absence of 2 neighbors	0.7	−211.9	Exergonic
(010)	In presence of 2 neighbors	29.9	−299.7	Exergonic
	In absence of 1 neighbor	20.5	−237.9	Exergonic
	In absence of 2 neighbors	7.1	−126.0	Exergonic

## Abbreviations

MD	Molecular Dynamics
CH	Portlandite (Ca(OH)_2_)
KMC	Kinetic Monte Carlo
ReaxFF	Reactive Force Field
LAMMPS	Large-Scale Atomic/Molecular Massively Parallel Simulator
metaD	Metadynamics
CVs	Collective Variables
TS	Transition State
VNL	Virtual Nano Lab
FES	Free Energy Surfaces (kJ/mol)
HFTN	Hessian-Free Truncated Newton Algorithm
NVT	Nose-Hoover Thermostat
NPT	Nose-Hoover Pressure Barostat
$\Delta G^{*}$	Free Energy of Activation Barrier (kJ/mol)
ΔG	Free Energy Change (kJ/mol)

## Appendix A

**Table A1:** Crystallographic data of Portlandite consisting of different crystal facets at 298 K.

Crystal Facet of Portlandite	The Dimension of the Sumulation Cell (Å^3^/10^−30^ m^3^)	No. of Atoms in the Simulation Cell
(001)	17.67, 19.94, 40.75	1307
(100)	20.75, 19.94, 38.42	1431
(1$\bar{1}$0)	20.75, 26.64, 46.41	2186
(010)	17.67, 20.75, 34.94	1155
